# CANreduce-SP—adding psychological support to web-based adherence-focused guided self-help for cannabis users: study protocol for a three-arm randomized control trial

**DOI:** 10.1186/s13063-022-06399-2

**Published:** 2022-06-22

**Authors:** J. I. Mestre-Pintó, F. Fonseca, M. P. Schaub, C. Baumgartner, M. Alias-Ferri, M. Torrens

**Affiliations:** 1 Addiction Research Group (GRAd), Neuroscience Research Program, Hospital del Mar Medical Research Institute (IMIM), 08003 Barcelona, Spain; 2 Department of Experimental and Health Sciences (CEXS), Universitat Pompeu Fabra, 08002 Barcelona, Spain; 3 Department of Psychiatry and Department of Pharmacology, School of Medicine, Universitat Autònoma de Barcelona (UAB), 08290 Cerdanyola del Vallès, Spain; 4 Swiss Research Institute for Public Health and Addiction, University of Zurich, Zurich, Switzerland; 5 Institut de Neuropsiquiatria i Addiccions, Hospital del Mar, 08003 Barcelona, Spain

**Keywords:** CANreduce, Cannabis use disorder, Psychological support, Cognitive behavioural therapy, Randomized controlled trial, Reducing cannabis, Self-help tool, Guidance, Adherence

## Abstract

**Background:**

Cannabis is the most-frequently used illicit drug in Europe. Over the last few years in Spain, treatment demand has increased, yet most cannabis users do not seek treatment despite the related problems. A web-based self-help tool, like CANreduce 2.0, could help these users to control their consumption.

**Methods:**

This study protocol describes a three-arm randomized controlled trial (RCT) comparing the effectiveness of three approaches, in terms of reducing cannabis use among problematic cannabis users, the first two treatment arms including the Spanish version of CANreduce 2.0 (an adherence-focused, guidance-enhanced, web-based self-help tool) (1) with and (2) without psychological support; and the third group (3) treatment as usual (TAU). Study hypotheses will be tested concerning the primary outcome: change in the number of days of cannabis use over the previous week, comparing assessments at 6 weeks and 3 and 6 months follow-up between groups and against baseline. Secondary outcomes related to cannabis use will be tested similarly. Mental disorders will be explored as predictors of adherence and outcomes. Analyses will be performed on an intention-to-treat basis, then verified by complete case analyses.

**Discussion:**

This study will test how effective the Spanish version of CANreduce 2.0 (CANreduce-SP) is at reducing both the frequency and quantity of cannabis use in problematic users and whether adding psychological support increases its effectiveness.

**Trial registration:**

This trial is registered with the Clinical Trials Protocol Registration and Results System (PRS) number: NCT04517474. Registered 18 August 2020, (Archived by archive.is https://archive.is/N1Y64). The project commenced in November 2020 and recruitment is anticipated to end by November 2022.

## Background

Cannabis is currently the third most frequently consumed drug in the world, after alcohol and tobacco. According to the latest report by the United Nations Office on Drugs and Crime, approximately 192 million people between 15 and 64 years old have used cannabis in the past year [1]. An increasing number of jurisdictions worldwide have legalized or are about to legalize cannabis. To date, however, it is hard to assess the influence these changes might have in many areas of interest, like the demand for treatment of cannabis use disorder (CUD), the changes’ impact on public health, and their legal ramifications [2, 3]. Nevertheless, it is noteworthy that both the number of people who use cannabis frequently and the overall frequency of the drug’s use have increased in all these jurisdictions since legalization, and more potent cannabis products are now more readily available in these markets [4, 5].

In Europe, the European Monitoring Center on Drugs and Drug Addiction (EMCDDA) estimated that 18 million Europeans from 15 to 34 years old (which is 15% of that age group) used cannabis in 2018 [6]. In Spain, the estimate was even higher, at 18.3%. The typical Spanish problematic cannabis user (defined as someone using cannabis at least 20 days monthly) is male (80%), under age 35 (70%), and single (70%), with a secondary education (72%) and currently working (49%), while smoking an average of three joints per day [7, 8]. There was, however, a sizeable gap identified during the Spanish general population survey called EDADES (Encuesta Sobre Alcohol y Drogas en España) between the estimated number of problematic cannabis users between 15 and 64 years old and the number in that same age group seeking treatment (6%) [9]. Cannabis users have a risk of addiction that ranges from 10% to 20% and this risk is increased by certain factors, like early-onset consumption, problematic consumption over the preceding 12 months [8], and higher product THC concentrations [10, 11].

The current acceptability of cannabis use as a personal option demands new regulations regarding its consumption and distribution. It also requires developing strategies that help convey to citizens the potential risks of cannabis use (short-term, long-term, and due to heavy use) and offer accessible and effective therapeutic approaches [12].

In Spain, a broad, diversified and readily-accessible public drug treatment network has been developed in recent decades [13]. According to the national health system, its underlying principles and goals focus on universality, free access, equity, and fairness of financing. Still, it should be noted that a high percentage of problematic cannabis users do not seek treatment anywhere (93%). Moreover, there is a lack of approved pharmacological treatments (despite a wide range of off-label treatments for CUD and cannabis withdrawal) and high attrition rates for most of these treatments [14]. Consequently, innovative intervention strategies are needed [9]. Today, young people are very familiar with the use of new technologies in almost all areas of life and the age group most likely to accept and benefit from new technologies used therapeutically [15].

The COVID-19 pandemic has brought a wide variety of unprecedented challenges, many of which appear to be affecting mental health and consumption patterns in cannabis users [16–19]. Therefore, it is essential for healthcare providers to use all the resources and therapies available to help users adjust to the problems caused by the pandemic. Recent meta-analyses [20–22] have assessed how effective internet interventions (OCIs) are at reducing cannabis use. However, these interventions are well known to have low adherence and follow-up rates [23].

The CANreduce intervention is an integrated, online, and self-guided treatment program designed to help cannabis users reduce their cannabis consumption. Developed by the Swiss Research Institute for Public Health and Addictions, it has been certified as safe by the European Union. It is considered a medical device under European Union guidelines 93/42/EWG and 2007/47/EWG. Randomized clinical trial (RCT) results were published in 2015, demonstrating CANreduce to be effective, though beset by an adherence rate that might be improved by creating internet-based applications responsive to all devices (phones, tablets, computers) and adding psychological support [24]. An updated, adherence-focused guidance enhanced version, CANreduce 2.0, has since been developed [25] and found to be effective [26].

For the current study, CANreduce 2.0 (an adherence-focused, guidance-enhanced, web-based self-help tool) [26] was translated into Spanish (CANreduce-SP) and its effectiveness with versus without psychological support compared, in terms of reducing cannabis use among problematic users. Adding psychological support (the option for program users to contact an e-coach to resolve doubts or any concerns they have regarding the program or process) is expected to optimize both adherence to and the effectiveness of the program. Previous studies have identified low demand for this type of support, but better results with similar support options, like chat sessions with a counsellor [24].

Study hypotheses will be tested concerning the main outcome: reduction in the number of days of cannabis use over the prior week, comparing assessments at baseline versus 6-week, 3-month, and 6-month follow-up. A priori study hypotheses are:

1. The CANreduce-SP program with psychological support (study arm 1) will be more effective than CANreduce-SP with no psychological support (study arm 2) at reducing cannabis use.
2. The CANreduce-SP program with psychological support (study arm 1) will be more effective than treatment as usual (TAU, study arm 3) at reducing cannabis use.
3. The CANreduce-SP program without psychological support (study arm 2) will be more effective than TAU (study arm 3) at reducing cannabis use.
4. Participants in study arm 1 will demonstrate better adherence than participants in study arm 2 over the 6-week intervention.

Secondary cannabis-related outcomes will be tested similarly, and frequently co-occurring mental disorders explored as predictors of adherence and outcomes.

## Methods/design

**Fig. 1:**
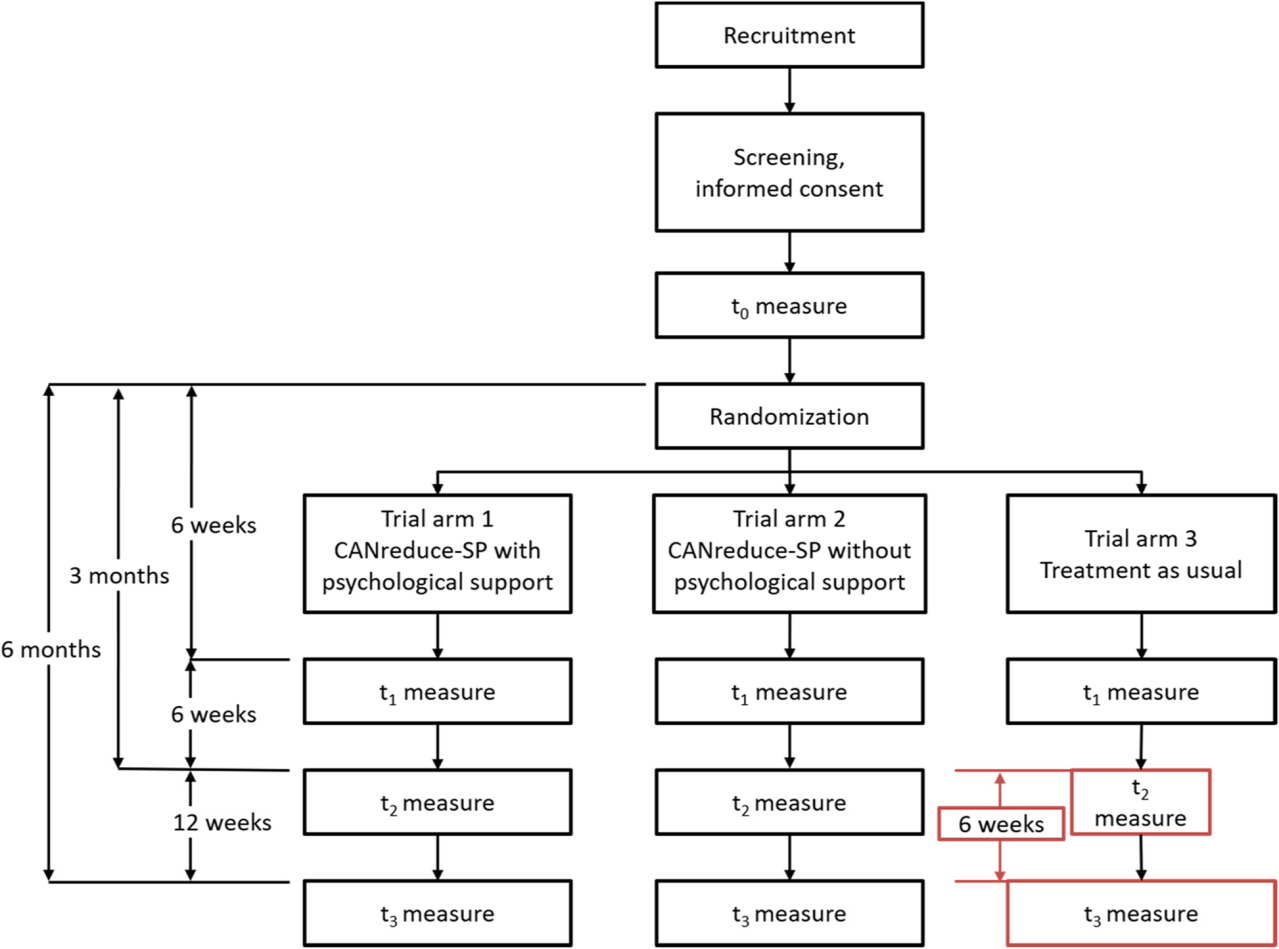
RCT study flowchart

The Spanish version of the adherence-focused, guidance-enhanced, web-based self-help program CANreduce 2.0 (CANreduce-SP) will be evaluated within a three-arm RCT, in which the effectiveness of CANreduce-SP with psychological support and CANreduce-SP without psychological support will be compared against each other and against treatment-as-usual (TAU) at reducing cannabis use in problematic users. Given that the study will only be partially single-blinded, the two active intervention arms will be neutrally described to subjects assigned to those arms to prevent participants from preferring one over the other. However, participants assigned to TAU will immediately be made aware of their group. As for study personnel, though any who directly provided support to program users cannot feasibly be blinded to subjects’ group assignments, all other study personnel (e.g. those involved in data collection, editing and analysis) will be. Co-occurring mental health symptoms will be screened with the rest of the primary and secondary outcomes during the baseline assessment (*t*_0_) to ensure that all potential participants meet study criteria and are eligible to be randomized and allocated to one of the three study conditions. Further assessments will take place 6 weeks after baseline, which is also the time when participants will have completed the 6-week program (*t*_1_), as well as at 3 months (*t*_2_), and 6 months (*t*_3_) follow-up (Fig. 1). Having 6 months of follow-up will allow us to examine the durability of any changes achieved with either of the two active interventions (study arms 1 and 2). For ethical reasons, we have elected not to ask study participants in the TAU group to wait a full 6 months for their final follow-up. Instead, we will grant them access to the intervention group without psychological support (study arm 2) 18 weeks after baseline—6 weeks after their 3-month evaluation (*t*_2_)—with the intention of assessing any response to the program they might have had at the 6-month follow-up assessment (t_3_). In this way, they will provide the study with a further, cross-over comparison.

This trial is registered with the Clinical Trials Protocol Registration and Results System (PRS) number: NCT04517474.

### Adaptations

The first required step for this study was to translate the CANreduce program from German into Spanish and culturally adapt its contents. The process was performed by external professional bilingual translators (German-Spanish, Spanish-German). After all adaptations, all text was reviewed and discussed by four mental health professionals (three psychiatrists and a psychologist) who are faculty within the Addiction Research Group at the Hospital del Mar Medical Research Institute (IMIM). Twelve cannabis users then accessed the first version of CANreduce-SP before study launch and were asked about the online interface (readability on different devices), content (appropriate, understandable, and use of appropriate colloquial language), and program (interest and value). Due to the COVID-19 pandemic, all these issues were discussed in an online focus group. Consensual changes were later implemented on the final version of the website.

### Recruitment of study participants

Recruitment started in November 2020 and may remain open until June 2022 to ensure that the estimated required sample size of 300 participants is met. Participants will be recruited through advertisements placed in Spanish cannabis social clubs, on university premises, and on online national search engines like Google Ads. Those who complete questionnaires upon program completion (*t*_1_) will be rewarded with a digital voucher worth €15. Those who complete the follow-up questionnaires at three (*t*_2_) and six (*t*_3_) months will be reimbursed with another €15 digital voucher for each these two time points. To enhance subject retention in the study, any participant who completes all the assessments will be rewarded with an extra €15 voucher.

### Registration and consent procedure

Applicants only are required to provide minimal personal data: e-mail address, phone number(s) (only if they need to be contacted to complete follow-up questionnaires), and limited primary sociodemographic data (age, gender, level of education, etc.). Registration for participation will be performed exclusively online. Before the register, each participant will be informed online about the study and will have the option to ask any questions they might have via e-mail before signing the online informed consent form. The information they will be provided is summarized in Table 1.

**Table 1:** Information provided to participants before they provide signed informed consent

1. Purpose, background, and an overview of the study
2. Inclusion and exclusion criteria (Table 2)
3. Description of the three treatment arms^a^ and their chances of being allocated to each one of these three arms
4. Financial aspects (no participation fee, compensation for participation)
5. Potential risks of participation and when to contact their general practitioner or a professional they can select from a medical advisory and emergency service list that will always be accessible to them, merely by clicking on the site’s Help icon
6. The inability of CANreduce-SP to replace face-to-face therapy for problematic cannabis use or abuse
7. Voluntary nature of their participation and their right to withdraw at any time without consequences, except for the loss of further compensation
8. Confidentiality and data protection (anonymity is ensured by not recording real names or postal addresses and by deleting e-mail addresses and phone numbers before statistical analysis and data archiving)
9. Protocol approval was granted by the ethics committee of the IMIM Hospital del Mar d'Investigacions Mèdiques after the committee had reviewed the study: num. 2019/8901/I.

^a^No details about how the two active treatment arms differ will be provided

**Table 2:** Inclusion and exclusion criteria and underlying rationale

**Inclusion criteria**	**Reasoning**
Informed consent via the Webform	To ensure adequate knowledge of the procedures and declare their consent
Minimal age of 18 years	To ensure a minimum age of participation
Cannabis use at least once weekly over the last 30 days	To include participants with less than daily cannabis use, increase the validity of results
At least once-weekly Internet access and a valid e-mail address	To ensure at least some access to the intervention
Good command of the Spanish language	To ensure that participants will be able to understand the information provided
**Exclusion criteria**	**Reasoning**
Participation in other psychosocial or pharmacological treatments for the reduction or cessation of cannabis use	To avoid confounding treatment effects
Current pharmacologically-treated psychiatric disease or any history of psychosis, schizophrenia, bipolar type I, substance use disorder, or significant current suicidal or homicidal thoughts	To avoid having subjects with these problems enter the study, since they could hinder the treatment’s effectiveness

Once participants have activated all checkboxes reaffirming essential study statements, they will be able to click the submit button, and informed consent will be assumed.

### Randomization and trial flow

Once participants have completed their baseline assessment (*t*_0_), they will be randomized by a computer algorithm in a 1:1:1 ratio into one of three parallel groups and will be registered automatically in the background database. Participants’ internet protocol (IP) address will be monitored to prevent multiple registrations from one user attempting to have more than a single account or be allocated to a different group than originally assigned. Participants assigned to the control condition (TAU/study arm 3) will be remitted to a list with the treatment centres nearest their postal code. After 3 months in TAU, participants will gain access to CANreduce-SP without psychological support. The four assessment points of the study are depicted in Fig. 1. All subjects will be asked about their potential for using some different treatment during their participation in the study. A reminder e-mail will be sent before each assessment. It will contain information about the value of their answers, the estimated length of the questionnaire (in time to complete), and compensation for each evaluation to enhance the response rate. The same e-mail will be sent if there is no answer within 2 days and again after 4 days. One week after the last e-mail, participants will be contacted by phone and will be offered the option of completing the follow-up questionnaire verbally with an interviewer. Participants who drop out will be asked to answer a few questions pertaining to the two the primary outcomes and why they have decided not to continue in the study. Inactivity will not be considered a dropout criterion, due to the breaks that participants of web-based interventions commonly take. It is expected that some of these users will re-join the program later. A participant’s active withdrawal from the study will be the only dropout criteria. If possible, all data already gathered from these participants will be analysed. Figure 1 depicts the flow of subjects through the study.

### Intervention

CANreduce 2.0 is an automated web-based self-help tool developed by the Swiss Research Institute for Public Health and Addiction (ISGF) to reduce cannabis consumption in problematic cannabis users [26]. It has three main components: a dashboard, a consumption diary, and eight modules designed to reduce cannabis use using the principles of motivational intervention, self-control practices, and cognitive behavioural therapy (see Table 3). Participants can study all the modules at their own pace and in whatever order they choose, though a specific order is advised.

**Table 3:** Brief description of the eight intervention modules (For further information, please see Amman [25] and Baumgartner [26])

**Module**	**Brief objective description**	**Therapeutic base**
**1 Introduction**	General overview and introducing the fictional companions	Motivational interviewing (MI) techniques [27]
**2 Identifying risky situations**	Identify personal risk situations and increase awareness of irrelevant decisions that could lead to cannabis use	Cognitive behavioural therapy (CBT) approach to relapse prevention [28]
**3 Working on needs**	Learn skills to strengthen social contacts, decrease possible ruminations, and develop healthier sleeping habits	Behavioural activation approach [29]
**4 Craving**	Learn strategies to deal with craving	CBT principles [30]
**5 Dealing with relapses**	Learn skills for relapse prevention	Cognitive behavioural therapy (CBT) approach to relapse prevention [28]
**6 Working on problems**	How to deal with common problems using new problem-solving skills	Social problem-solving approach [31]
**7 Saying “No”**	Strengthen refusal skills	CBT principles [30]
**8 Preserving achievements**	Review the work done and create a personalized list of points to secure achievements made	MI techniques [27]

Since CANreduce 2.0 is considered a medical device, because of the European Union guidelines 93/42/EWG and 2007/47/EWG, its conformity has been assessed, and all potential risks have been evaluated.

### Active study arms

**Fig. 2:**
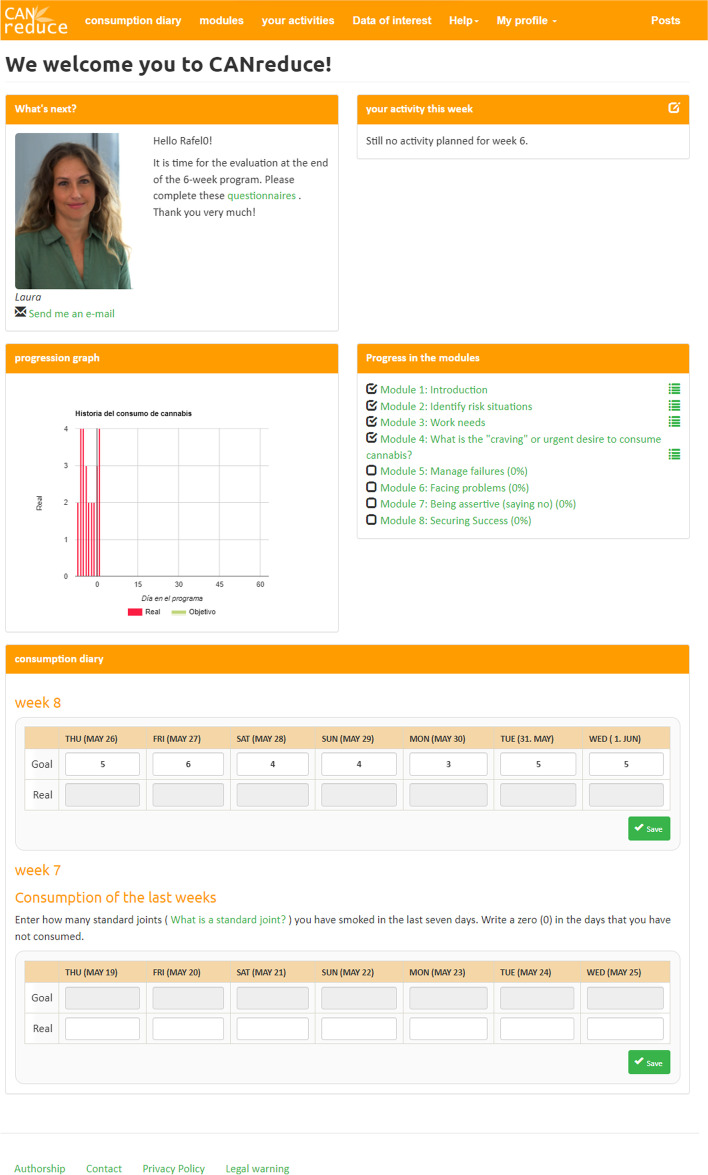
Dashboard for study arms 1 and 2 (translated from Spanish to English for publication purposes only)

Both active study arms have the enhanced version CANreduce 2.0 with social presence that is described in Amann and Baumgartner [25, 26]. Psychological support (feedback on demand) is only present in study arm 1 and includes offering participants the opportunity to contact an e-Coach via the platform’s internal messaging system (Fig. 2) or by e-mail. As such, they can receive psychological support when needed, though it will only take place on their initiative. Within two workdays, the participants will receive personalized written feedback. Both active treatment arms (study arms 1 and 2) will have access to the following elements of the CANreduce-SP program (for a detailed description of the content of each element, please review Amann and Baumgartner [25, 26]):

#### Welcome page

Before accessing the restricted area, users are introduced to the welcome page, which has a short, written description of the study and an introductory video in which the e-coach Laura introduces herself and gives a general overview of CANreduce highlights and objectives. Both the written description and the video aim to motivate visitors to ask for more information about the study and how to enrol by clicking the inscription button that leads to the registration and consent procedure explained above.

#### Dashboard

The dashboard (see Fig. 2) works as the main page of the program. All the practical information is displayed clearly and briefly, including a chart with expected and actual consumption within the program, indicators of up-to-date progress on the intervention modules, and a weekly calendar to record cannabis consumption over the preceding week. The Message button at the top right of the screen only appears in the intervention group with psychological support (arm 1).

#### Self-help intervention modules

**Fig. 3:**
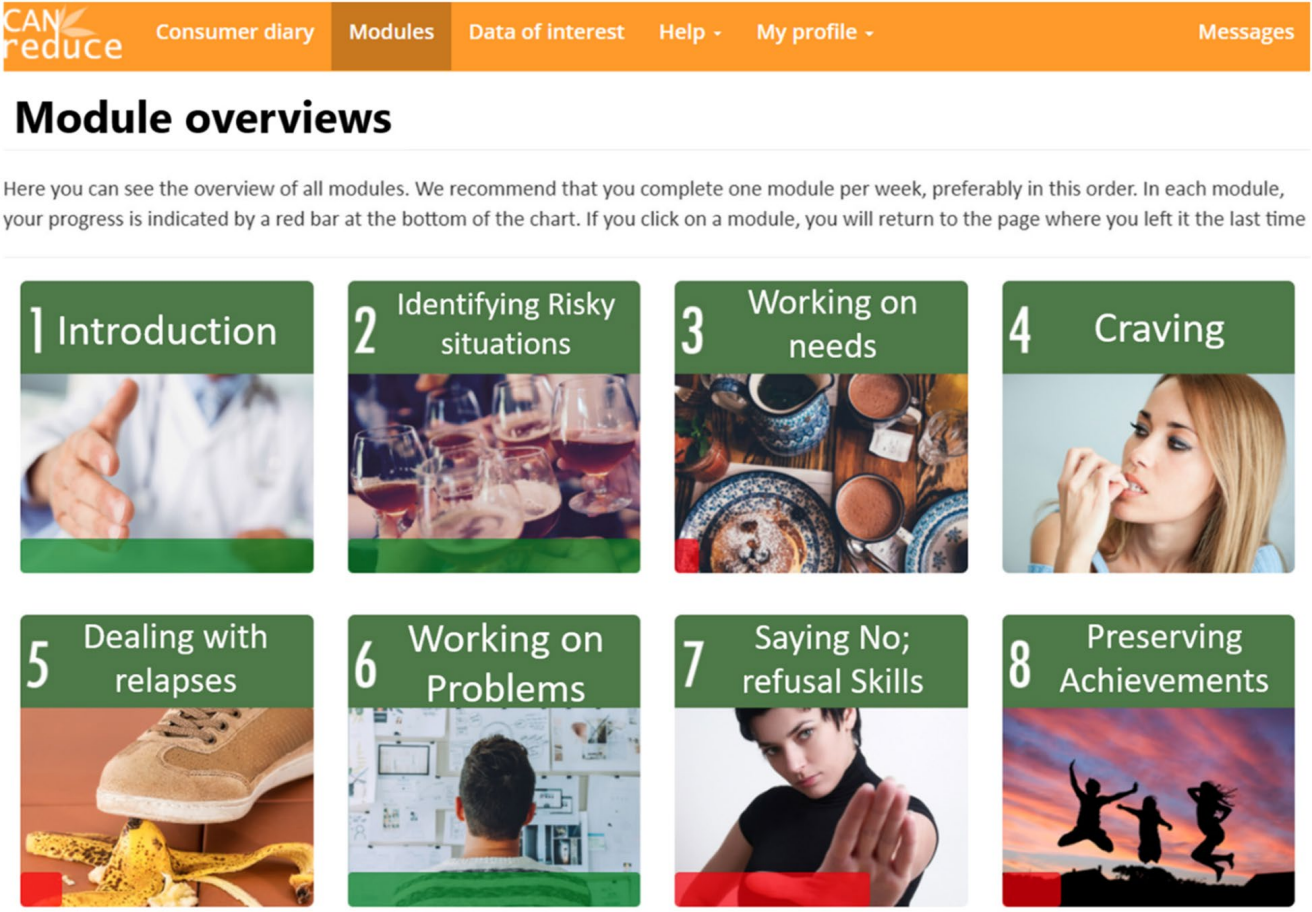
Module overviews (translated from Spanish to English for publication purposes only)

The intervention modules (Table 3) are the same for both active intervention groups (arms 1 and 2). They are accessible from the very beginning, both on the intervention website's main menu page (see Fig. 3) and on the dashboard (see Fig. 2). It is recommended that users complete the modules sequentially at a pace of one or two per week; however, participants are free to proceed at their own pace, complete modules in whatever order they please, and repeat any modules they consider particularly helpful or essential.

#### Fictional companions

Six fictional companions were created to accompany users during the program. Certain questions have been identified that are commonly asked by users at crucial points in each module. These companions are intended to help participants overcome their doubts dealing with these questions and situations, by providing them examples of what they themselves did, thereby encouraging study participants to reflect and share their own thoughts and experiences.

#### Consumption diary

All users are required to define their standard joint to activate the registration process. The configuration includes (1) the type of cannabis (indoor, outdoor, resin); (2) if it is blended with tobacco or not; and (3) the amount they use ranging from 67 to 500 mg. All possible configurations are represented with a photo to provide automatic visual feedback to fit the user’s standard joint. The individual’s standard joint cannot be changed once this step is completed. Creating this personalized standard unit helps users to record their target of weekly consumption and actual consumption over the preceding week.

#### Extra elements

The program contains several complementary resources through the menu on the top bar. In “Data of interest,” neutral information about cannabis is provided, from its mechanism of action and effects on the brain and consciousness to issues related to Spanish legislation. In the section “Your activities,” users can plan weekly events as alternatives to consumption situations. They can then compare their initial expectations with the experience itself, thereby potentially encouraging them to repeat positive experiences. In the section “Help,” participants can access both the CANreduce-SP instructions and the specific resources for urgent help to find out what to do in case a crisis arises (nearby care centres and recommendations to overcome moments of crisis). Finally, in the “My profile” menu, they can access their annotations and summarize the personal contributions and strategies that each user has established in the program’s modules.

#### Automated motivational feedback

Active intervention group participants are encouraged to fill out their consumption diary at least once every week via a reminder e-mail. This process promotes goal-setting (Mohr’s accountability factor) and tests and improves self-efficacy without the self-deception usually seen in a professional therapy setting. Additional e-mails can be sent out automatically or triggered by an investigator if certain conditions arise (e.g. not completing the consumption diary, stalling, increasing cannabis consumption, or underutilizing program resources). Regarding automatic e-mails, the only difference between the active arms is the addition of the paragraph encouraging participants to initiate contact for psychological support (feedback on demand) that was added to study arm 1 (see Table 4).

**Table 4:** First-week e-mail example (translated from Spanish to English for publication purposes only)

Study arm 1 and 2 (paragraph in italics ONLY appears in study arm 1)
Hello {participant’s name }! You've made it through the first week. Congratulations! I hope you've been well these past few days. Your second week starts tomorrow. Log in today or late in the afternoon at https://www.CANreduce.es and record in your user diary how much cannabis you have consumed in the last few days. You can also add when and how much cannabis you would like to use next week. I take this time to encourage you to start another one of the eight modules. If you do the modules, you will get the most out of the course and achieve your goals. *Remember that if you need my opinion about your proposals or ideas or want me to help you with any of the exercises, you only have to send me an e-mail explaining what you want. Just indicate which exercise on which you need my help or opinion. I will answer you within 48 hours on work days.* Go for it, and happy week! Your e-coach Laura

#### Psychological support

Several elements of human support increase adherence (e.g. accountability to a coach who is considered trustworthy) [32]. As explained by Mohr\s supportive accountability, human support may be provided by a wide range of trained individuals [33]. The feedback received does not directly influence the treatment’s effectiveness but is more a strategy to highlight human support during the process. The goal is to increase confidence in the trainer and a sense that the trainer is concerned with the participant’s interests [33, 34]. This strategy increases the perception of legitimacy that would encourage people to respond to adherence demands actively. We provide automated human support through e-mails, videos, and reminders to both active groups. Besides this, we offer psychological support (feedback on demand) via personal contact with the e-coach to ask questions or express concerns via the internal message system or e-mail to increase this human support effect and evaluate the level of demand for this service.

#### Control condition

The control condition will be treatment as usual (TAU). Participants will be informed about this group's relevance, their path in the study (Fig. 1) and the link to the Plan Nacional Sobre Drogas (National Drug Plan) web page to find information about the drug treatment centre nearest their place of residence. During the 3-month assessment at (*t*_2_), as with the two active intervention groups (study arms 1 and 2), TAU group participants will be asked if they have used other treatments during their study participation and what they were. Then, they will be reallocated into intervention group 2 (study arm 2) to access all the CANreduce resources, except psychological support, and will have their last assessment (t_3_) after this 6-week program.

#### Technical specifications

CANreduce-SP is based on the Swiss Program CANreduce 2.0 [25, 26], which was programmed and developed using the content management system Drupal 7 and a MySQL database. All access to the study website CANreduce.es [35] is encrypted and password-protected using the secure sockets layer (SSL) protocol. Only participants will have access to any data they have previously entered online on their own. Data validation and mandatory fields on website boxes are enabled to prevent logical errors or data missing in the study database. The webpage has a responsive design to adapt to any portable device automatically. Completing the registration process requires potential users to have a valid e-mail and activate checkboxes pertaining to the essential ethical and responsible aspects of the study (see Tables 1 and 2). All data will be stored on a server hosted by an external provider that meets the IT security outsourcing regulations of the European GDPR 2016/976. Final data will be extracted from the database using Drupal and phpMyAdmin. The data will then be stored at the principal investigator’s institution on local computers for further processing and local file servers for archiving. All participants’ personal data will be deleted after their participation in the study and will never be published or presented at scientific meetings. Anonymized study data will be available upon request. The investigator affirms and upholds the principle of every participant's right to privacy and that all personnel involved in the study will comply with applicable privacy laws.

### Measurements

All data will be retrieved online. Sociodemographic data will include subject gender, age, autonomous community, education level, and work status. The primary outcome will be the number of days of cannabis use over the preceding seven days, according to the Time-Line-Follow-Back (TLFB) method [36, 37]. The TLFB is a calendar used in many studies to obtain estimates of the quantity of daily drug use (alcohol, cannabis, cocaine…). It is broadly recommended to be used for outcome evaluation and treatment planning purposes.

Secondary outcomes will include the number of days of cannabis use over the last 30 days, again using the TLFB method, and the number of standardized cannabis joints [24] consumed in the previous week. The presence of a cannabis use disorder (CUD) will be measured by the CUDIT-R (Cannabis Use Disorder Identification Test-Revised, CUDIT-R) [38], a validated and quick-to-use self-administered questionnaire with eight items that has been found to exhibit excellent psychometric properties. Questions 1–7 are scored on a 0–4 scale (range 0–28), and question 8 is scored 0, 2, or 4 (total range score 0–32). Participants scoring 8 or more may have hazardous cannabis use, while scoring 12 or more indicates potential cannabis use disorder. The Severity Dependence Scale (SDS) [39, 40] was added to assess the severity of cannabis use disorder. It has five items scored on a 4-point scale (0-3) with a total range score of 0 to 15, where higher scores denote worse severity. The Alcohol Use Disorders Identification Test (AUDIT) [41, 42] was developed by the World Health Organization (WHO) to assess alcohol consumption and alcohol-related problems. Each of the 10-items is scored 0–4, with a total score ranging from 0 to 40. Scores of 8 or more suggest hazardous or harmful alcohol use. Anxiety and depression are assessed with PROMIS (Patient-Reported Outcomes Measurement Information System). PROMIS is a set of person-centred measures that evaluate and monitor physical, mental, and social health in adults and children. It can be used within the general population and among individuals living with chronic disorders. It was developed and has been validated with state-of-the-science methods to be psychometrically sound and to transform how life domains are measured. Both the PROMIS Emotional Distress - Depression - Short Form 8b and the PROMIS Anxiety 8a - Adult v1.0 have eight items; each scored using a Likert scale ranging from 1 to 5, with total scores ranging from 8 to 40. [43, 44]. In both instruments, higher scores imply worse outcomes. Quality of life is assessed with EQ-5D-5 L, known for being an excellent, self-administered, health-related quality of life questionnaire [45]. The scale measures quality of life across five domains: mobility, self-care, usual activities, pain/discomfort, and anxiety/depression. Total scores range from less than 0, where 0 is the value of a health state equivalent to dead; negative values represent values worse than death (i.e. a life that an individual considers not worth living due to, for example, intractable, intolerable pain), and 1 indicates full health. The second part of the questionnaire consists of a visual analogue scale (VAS) on which the patient rates his/her perceived health from 0 (worst imaginable health) to 100 (best imaginable health).

At the 3-month follow-up assessment (*t*_2_), any adverse effects will be identified using Rozental’s Negative Effects of Internet Interventions [46]. Finally, all participants will be asked if they have used any treatment other than CANreduce-SP during their participation in the program. If so, we will ask them to describe the treatment they used. Data on webpage usage (i.e. number of completed modules, time spent on the website) will be collected as an indicator of treatment adherence. These data could help to identify ways to optimize CANreduce-SP to decrease attrition rates.

### Sample size calculation

Anticipating that a Cohen’s *d* of 0.40 would be realistic to determine effect size differences on the primary outcome between groups, based on previous study experiences [23–26], a sample size of *n* = 100 in each study group would have 80% power on two-tailed *t*-test calculations performed using G*Power software (Faul, Kiel) and an alpha error of 5%. Given an average attrition rate of 50% in online studies involving cannabis misusers [26], our final adjusted sample size is *N* = 450.

### Data analysis

All analyses will be performed on an intention-to-treat basis and verified by complete case analyses. Imputation procedures will be chosen based on missing data patterns (e.g. using the MICE multiple imputation function in the statistical software package R and between 20 and 40 imputed datasets, depending on the extent of missing data, in accordance with suggestions made by Graham [47]). Moreover, study dropout analysis will be performed comparing participants who completed the follow-up survey against those who did not. Cohen’s *d* will be determined, with the significance threshold for all analyses defined as alpha = .05. The primary outcome—number of days of cannabis use over the preceding 7 days, estimated using the Time-Line-Follow-Back method—will be analysed by means of linear regression models with baseline characteristics and group allocation as predictors. Secondary outcomes will be analysed accordingly.

Since this will be a low-risk intervention, there will be no independent data monitoring committee. Study data will be analysed by the lead and the principal investigator after completion of data acquisition. Planned analyses are described in this section. There will be no formal stopping rules since we do not anticipate problems detrimental to participants. Data collection will be stopped when at least one hundred and fifty participants will be included in each group. The lead investigator and the principal investigator will make the final decision to terminate the data collection.

### Composition of the coordinating centre and trial steering committee

The steering committee consisting of all lead and principal investigators of the two research centres will have virtual meetings to address goals and progress of the project monthly for 6 months and later when needed. The lead investigator of the present RCT trial was responsible for the study design, prepared the protocol, and will organize weekly virtual or presential meetings with the trial management group. In these meetings, the trial management group will report on problems and progress of ongoing data collection. The trial management group will consist of 2 research assistants (psychologist) responsible for Google Ads review, who will answer to the participants’ emails (general questions or psychological support), sending the SMS reminders and helping participants to fulfil the follow-up questionnaires by phone. The lead investigator together with the principal investigator will decide on trial termination and will write up study results for publication.

### Frequency and plans for auditing trial conduct

Members of the steering committee not directly involved in the conduction of the study will perform an annual audit, to evaluate compliance with the study protocol.

### Safety

No drugs will be administered in this study, and potential risks related to withdrawal symptoms (i.e. sleep problems, mild depressive symptoms, or craving) are expected to be minimal. Furthermore, these symptoms will be addressed in the psycho-educational modules of the intervention. The medical device (i.e. the self-help tool) was determined to be of very low risk during its European conformity certification. All participants will have access to a help page with instructions on how to deal with common situations and symptoms and possible actions if their situation becomes unbearable. These instructions include a link with a list of all professional healthcare providers in Spain who can be sorted by postal code or address.

## Results

The study will be conducted following the ethics board–approved protocol and the principles stated in the current version of the Declaration of Helsinki, the CONSORT eHealth [48] and SPIRIT Guidelines [49] for studies on medical devices, the European Directive on medical devices 93/42/EEC, and the ISO Norm 14155 and ISO 14971. The study was approved by the ethics committee of the CEIm-Parc de Salut Mar/IMIM-Hospital del Mar (Nr. 2019-8901-I) and is registered at Clinical Trials, traceable as NCT04517474. Results will be published in a scientific peer-reviewed journal. A lay-person-friendly summary of trial findings will be written and sent to all participants who have requested so when they registered.

## Discussion

### Expected principal findings

This study will provide the Spanish version (CANreduce-SP) of the Web-based self-help intervention program (CANreduce 2.0). It also will assess the effectiveness of CANreduce-SP with psychological support (e-mail contact with an e-coach) and without this personalized support. We expect that providing the “human element" features (e-coach videos and automated e-mails with a personalized feel) in both active intervention groups (study arms 1 and 2) will increase general program effectiveness. Furthermore, the group with psychological support will perform even better. As in previous studies, we expect very few actual e-mails to be sent by subjects seeking answers or help. In the end, we will analyse the cost-effectiveness and utility of this measure and its impact on outcomes.

The results will give us information on the efficacy of the CANreduce 2.0 program in the Spanish population. They will allow us to establish the costs and workload involved providing psychological support and its impact on adherence. This point is highly relevant, given that the Spanish-speaking population is huge globally. An autonomous tool could easily be adapted to any Spanish dialect; however, the need for support should be conveyed differently and linked to different countries’ health systems resources.

### Limitations

The first study limitation is related to inclusion criteria, in that cannabis users currently receiving other treatments to reduce cannabis consumption will be excluded. Second, all measurements will be self-reported and have not been validated in an online context, though they have largely been validated in other research and clinical settings. Third, as discovered in previous studies [24, 26, 50], we expect relatively high dropout rates. Finally, web-based studies tend to have low adherence rates, because of the distant nature of such interventions.

### Trial status

This trial is registered with the Clinical Trials Protocol Registration and Results System (PRS) number: NCT04517474. Registered 18 August 2020, https://clinicaltrials.gov/ct2/show/NCT04517474 (archived by archive.is https://archive.is/N1Y64). The project commenced in November 2020 and recruitment is anticipated to end by November 2022.

## Data Availability

The anonymized datasets analysed during the current study and statistical code will be available from the corresponding author on reasonable request, as is the full protocol and informed consent form.

## Abbreviations

RCT: Randomized controlled trial
TAU: Treatment as usual
CUD: Cannabis use disorder
EMCDDA: European Monitoring Center on Drugs and Drug Addiction
EDADES: Encuesta Sobre Alcohol y Drogas en España
OCIs: Internet Interventions
ISGF: Swiss Research Institute for Public Health and Addiction
CBT: Cognitive behavioural therapy
MI: Motivational Interviewing
TLFB: Time-Line-Follow-Back
CUDIT-R: Cannabis Use Disorder Identification Test-Revised
SDS: Severity Dependence Scale
AUDIT: Alcohol Use Disorders Identification Test
WHO: World Health Organization
PROMIS: Patient-Reported Outcomes Measurement Information System
VAS: Visual analogue scale
